# Supplementary material for: Learning curve and surgical outcome of robotic assisted colorectal surgery with ERAS program
Source: Sci Rep. 2022 Nov 29;12:20566. doi: 10.1038/s41598-022-24665-w (PMC9709162; doi:10.1038/s41598-022-24665-w)
Supplement: Supplementary file 1 — Supplementary Information 1. [file 41598_2022_24665_MOESM1_ESM.zip › LSC Raw data-Ñ[▒K/120/N_ñw╝╨░O▒Kñσ.pdf]

W55 / W55

臺中榮民總醫院
手術室護理記錄

[redacted]

基本資料

- 室別： 開刀房-18室，手術科別： CRS，手術類別： 常規，日期： 2021/03/12
- 進入時間： 0745 ； 麻醉開始時間： 0745 ； Time Out時間： 0930 ； 劃刀時間： 0935 ； 手術結束時間： 1400 送出時間： 1425 ；
- 麻醉方式： GE
  診斷：SIGMOID COLON Ca
  手術名稱：Laparoscopic Anterior resection and anastomosis - malignant
  手術局部用藥：NIL
  術前預防性抗生素：
  - 抗生素名稱一： Veterin 1GM IVD由麻醉護理人員給予
  - 時間： 0920
  - 上次簽章: 吳貞儀

  ，追加預防性抗生素：
- 手術醫師： 蔣鋒帆
- 麻醉醫師： R 曾含芳
-
  - 刷手護士A ： 林怡廷，時間： 0745，接替刷手一： 吳貞儀，時間： 1230
  - 流動護士A ： 吳貞儀，時間： 0745，接替流動一： 何宛臻，時間： 1200，接替流動二： 紀玉娟，時間： 1230
- 更多接替及流動護士： 接替流動三： 林怡廷，時間： 1300

皮膚狀況

A-V Shunt： 無
術前： 完整
記錄者： 上次簽章: 吳貞儀
術後： 不完整

- 7. 壓紅可白

部位(術後)：

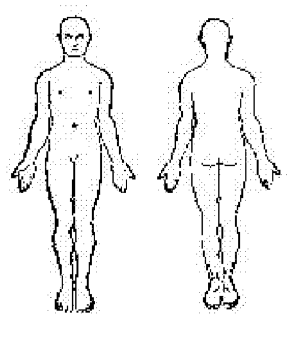

右下唇1*1cm水泡+左鼻2*2cm紅可白+雙肩胛5*5cm紅可白+薦骨5*5cm紅可白

記錄者： 林怡廷

手術準備

手術臥式 單一手術臥式

-
  - 手術臥式： 截石臥式
  - 使用附件： 溫毯 38 ℃，頭圈，腳架，其它： 頭架、雙腳下silicon pad
  - 皮膚準備： 10%Aq Betadine，Hibiscrub，2% Chlorhexidine
  - 電燒： 使用電燒: 電極片位置： 右大腿，記錄者： 上次簽章: 吳貞儀

止血帶T： 無

植入物

- 植入物名稱/號碼/數量/部位： COVIDIEN ENDO GIA 60mm Medium Thick*2/小腸
  EEA 29mm/S-colon

, 記錄者： 上次簽章: 林怡廷

標本送檢

- 細菌培養 一般，採取部位: 1. Ascites，數量： 1
- 細胞學 採取部位: 1. ACSITIS，數量： 1
- 冰凍切片 無
- 病理標本
  組織名稱一： R-S COLON
  ,
  組織名稱二： IMA root
  ,
  組織名稱三： distal cut end
- 截肢 無

標本交付 標本： 3 袋，標本： 0 盒，標本： 0 瓶，細胞學： 0 管，Culture Tube： 1 管，被交付醫師： R 劉佳樺，

- 記錄者： 上次簽章: 林怡廷
- 時間： 1400

傷口術後敷料

傷口敷料： 傷口敷料： 無

- 請於圖上標示部位：

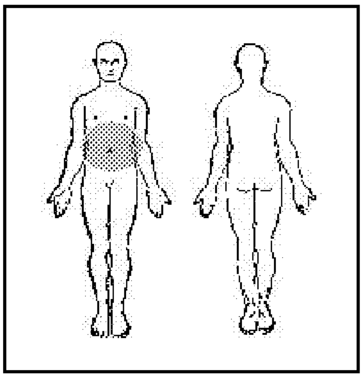

- 傷口敷料： 軟性敷料

導管

導管	天數	導管資訊	插入日期	插入時間	更換日期	拔除日期	拔除時間	拔除原因	預計拔除/更換	預計拔除/更換日期
Foley(1)	1	號碼:16 Fr.; 部位:膀胱;	2021/03/12	0830						
Sump NG Tube(1)	1	號碼:16 Fr.; 深度:65cm; 部位:胃;	2021/03/12	0900						
CVC(1)	1	號碼:8fr ; 深度:15cm; 部位:subclavian (右); 象限:RU;	2021/03/12	0910						
Epidural PCA(1)	1	部位:硬脊膜下;	2021/03/12	0830						

用物計數

- 手術中出血量： 少 c.c.
- 手術中尿量： 300 c.c.
-
  - 其它： 無

用物計數： 多切口同時縫合

- 1.器械： 正確，2.縫針： 正確，3.刀片： 正確，4.紗布： 正確，5.腹部墊： 未使用，6.紗球： 未使用，7.PEANUTS ： 未使用，8.紗條： 未使用，9.絲帶： 未使用，10.針頭： 未使用，11.Vessel loop： 未使用，12.Boots： 未使用，13.Jelco： 未使用，14.棉條： 未使用，

- 第一次：
  - 刷手護士 ： 吳貞儀
  - 流動護士： 上次簽章: 林怡廷
  - 時間： 1330

,

- 第二次：
  - 刷手護士 ： 吳貞儀
  - 流動護士： 上次簽章: 林怡廷
  - 時間： 1340

,

- 第三次：
  - 刷手護士 ： 吳貞儀
  - 流動護士： 上次簽章: 林怡廷
  - 時間： 1350

備註

備註： R6 NO.1 腹腔鏡

- 記錄者： 上次簽章: 吳貞儀
- 時間： 1018

填寫護理人員_林怡廷_

正式摘要

W55 / W55

doctorRank:B
